# Immediate and Delayed Hypersensitivity Reactions to Antibiotics: Aminoglycosides, Clindamycin, Linezolid, and Metronidazole

**DOI:** 10.1007/s12016-021-08878-x

**Published:** 2021-12-15

**Authors:** Michelle Dilley, Bob Geng

**Affiliations:** University of California San Diego and Rady Children’s Hospital, San Diego, CA USA

**Keywords:** Hypersensitivity reaction, Drug allergy, Aminoglycosides, Clindamycin, Linezolid, Metronidazole

## Abstract

Hypersensitivity reactions including IgE-mediated and delayed cell-mediated reactions to aminoglycosides, clindamycin, linezolid, and metronidazole are rare. For aminoglycosides, allergic contact dermatitis is the most frequent reaction for which patch testing can be a useful step in evaluation. For clindamycin, delayed maculopapular exanthems are the most common reactions. There are case reports of clindamycin associated with drug rash with eosinophilia and systemic symptoms (DRESS), acute generalized exanthematous pustulosis (AGEP), acute febrile neutrophilic dermatosis, and symmetrical drug-related intertriginous and flexural exanthema (SDRIFE). For linezolid, cases of hypersensitivity were exceedingly rare and included urticaria, angioedema, anaphylaxis, delayed rashes, and DRESS. For metronidazole, only rare cases were found across a broad spectrum of reactions including allergic contact dermatitis, fixed drug eruption, angioedema, anaphylaxis, serum sickness-like reaction, SJS/TEN, AGEP, SDRIFE, and a possible case of DRESS. IgE-mediated reactions and anaphylaxis to these types of antibiotics are uncommon, and reports of skin testing concentrations and desensitization protocols are largely limited to case reports and series. Non-irritating skin testing concentrations have been reported for gentamycin, tobramycin, and clindamycin. Published desensitization protocols for intravenous and inhaled tobramycin, oral clindamycin, intravenous linezolid, and oral and intravenous metronidazole have also been reported and are reviewed.

## Introduction

Aminoglycosides, clindamycin, linezolid, and metronidazole cause hypersensitivity reactions relatively infrequently when compared with beta-lactam antibiotics and sulfonamides. Due to the infrequent nature of these drug allergies, a review of the current literature on hypersensitivity reactions to these antibiotics is lacking. This review covers the most commonly reported hypersensitivity reaction types including epidemiological data and published evaluation/diagnostic strategies and desensitization protocols for each of these antimicrobial groups. 

## Aminoglycosides

### *streptomycin, gentamicin, tobramycin, neomycin, amikacin, kanamycin, plazomicin, paromomycin*

### Overview

#### **Structure**:

**Fig. 1:**
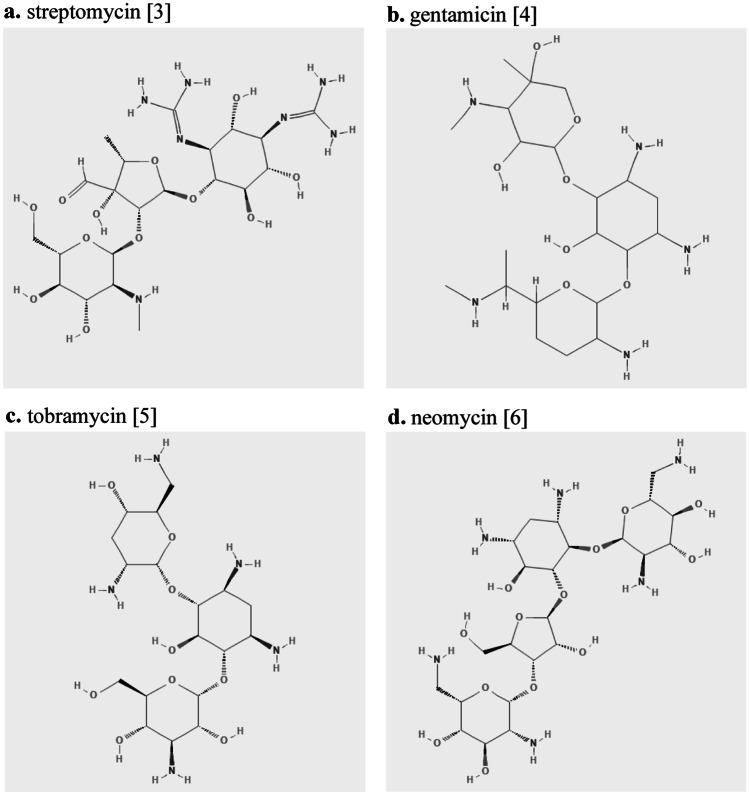
Chemical structure of aminoglycosides. Structural groups: **a** streptidine; **b**, **c**, and **d** deoxystreptamines

 Aminoglycosides have a hexose ring with amino group substituents to which various amino sugars are attached via glycosidic linkages [1, 2]. Aminoglycosides are classified into two main structural groups including the streptidine group consisting of streptomycin and the deoxystreptamine group consisting of gentamicin, tobramycin, neomycin, amikacin, kanamycin, plazomicin, and paromomycin [1–6] (Fig. 1).

#### **Mechanism**:

 Aminoglycosides are bactericidal and inhibit protein synthesis by binding to the 30S ribosomal subunit [2].

#### **Indications**:

 Aminoglycosides are particularly useful for infections caused by Gram-negative bacilli including Enterobacteriaceae, Pseudomonas species, Acinetobacter species, and Haemophilus influenzae*.* When combined with other agents, aminoglycosides can have effects against gram-positive organisms such as Staphylococcus aureus, Streptococci, and Enterococci [2]. Some aminoglycosides, particularly streptomycin and amikacin, are also active against mycobacteria [1, 7]. In addition to oral and intravenous (IV) administration for systemic infections, many aminoglycosides come in other forms. For example, gentamicin and tobramycin are available in topical forms to treat ophthalmic, otic, and skin infections [7]. Tobramycin and amikacin are available in inhaled forms, such as nebulized solutions and inhalation powders or suspensions to treat pulmonary infections [7].

#### **Known Pharmacologic Adverse Reactions**:

 The primary toxicities of aminoglycosides are nephrotoxicity and ototoxicity [1]. Given the need for serum drug concentration monitoring and the availability of alternative and less toxic agents, the widespread use of aminoglycosides is limited [2].

#### Hypersensitivity Reactions

Allergic reactions to aminoglycosides occur infrequently but are most commonly found to cause allergic contact dermatitis [8]. Other cutaneous reactions as well as systemic reactions including anaphylaxis have been published in case reports [8].

##### Allergic Contact Dermatitis

Allergic contact dermatitis, a type IV hypersensitivity reaction, is the most frequent reaction associated with this class of antimicrobials [8]. Aminoglycosides such as neomycin, tobramycin, and gentamicin are widely used in the USA for their topical application as creams, ointments, eye, or ear drops likely contributing to sensitization. Overall, aminoglycosides, including neomycin, tobramycin, and gentamicin, were found to be the most frequent topical ophthalmic medications to cause allergic contact dermatitis [11, 12]. The occurrence of positive patch test reactions to aminoglycosides increases with age [1].

Neomycin is one of the most common sensitizers among topical medications in general [1, 9, 10]. Neomycin-induced contact dermatitis occurs especially in patients with atopic eczema, chronic conjunctivitis or otitis, leg ulcers, and long-term cutaneous use of the drug [1]. The prevalence of neomycin hypersensitivity has increased with its increased accessibility. Neomycin contact dermatitis reactions are more prevalent in the USA ranging from 7 to 13% compared to Europe ranging from 1.2 to 5.4% [14]. Contact reaction rates for neomycin have decreased in Canada and are now similar to Europe, a trend likely influenced by the reduced availability of over-the-counter and prescription neomycin topical products in Canada [15].

Gentamicin in eye and ear drops is implicated in contact dermatitis but less often than neomycin [11]. Corazza et al. describe a case of a patient previously sensitized to topical neomycin with widespread eczematous dermatitis from gentamicin cream with confirmed positive patch testing to gentamicin [13]. Many of the reports of localized and systemic reactions to aminoglycosides occurred in patients with known prior exposure to some dosage form of an aminoglycoside, usually a topical dosage form, likely causing sensitization [7].

##### IgE-Mediated Reactions

Although there is no definitive evidence of IgE-mediated immediate hypersensitivity to aminoglycosides [1], there are a couple cases of patients experiencing immediate generalized rashes following administration of IV aminoglycosides [16–18]. Two reported cases of hypersensitivity reactions to inhaled tobramycin occurred in pediatric patients with cystic fibrosis who had previously experienced immediate generalized urticarial reactions to IV tobramycin or IV gentamicin [16, 17]. The rashes resolved when discontinuing the IV aminoglycosides but immediately recurred when these patients were subsequently administered inhaled tobramycin [16, 17]. Another case involved a patient with cystic fibrosis developing recurrent eosinophilia and severe persistent bronchospasm after repeat administration of inhaled tobramycin. This patient also had similar symptoms when later administered IV tobramycin [18].

There have been a few reported cases of possible anaphylaxis to aminoglycosides. Connolly et al. describe a case of immediate urticaria, hypotension, and loss of consciousness following IV gentamicin for surgical prophylaxis [19]. Christiansen el at describes a patient previously exposed to gentamicin in bone cement with hypotension, generalized erythema, and angioedema a few minutes after IV gentamicin also used for surgical prophylaxis [20]. Henao et al. report a case of urticaria, angioedema, and respiratory distress after multiple days of IM gentamicin for a superinfected third-degree burn [21]. Jung et al. describe a patient with a history of generalized urticaria within 1 h after administration of an IV aminoglycoside who experienced initial ear-itching and facial erythema followed by generalized urticaria, dyspnea, chest discomfort, and dizziness 10 min after intradermal testing with streptomycin at a concentration of 1 mg/mL [22].

##### Other Hypersensitivity Reactions

Other cutaneous manifestations like urticaria and DRESS have been reported [23, 24]. Añíbarro and Seoane describe a case of a patient who experienced immediate urticaria following topical nasal application of neomycin and who had positive skin prick test (SPT) to neomycin sulfate [23]. A case of DRESS weeks after starting amikacin was described and consisted of a maculopapular rash, facial edema, fever, hypereosinophilia, transaminitis, and coagulopathy [24].

##### Cross-Reactivity

Cross-reactivity among aminoglycosides is common due to similarities in chemical structure (Fig. 1). Cross-reactivity approaches 50% or more in the deoxystreptamine group (consisting of gentamicin, tobramycin, neomycin, amikacin, kanamycin, plazomicin, and paromomycin) [1]. For patients found to be allergic to neomycin, 65% had a cross-allergic reaction to tobramycin on patch testing [7]. Therefore, all deoxystreptamine-containing aminoglycosides are contraindicated if a patient has a known hypersensitivity to another deoxystreptamine-containing aminoglycoside [1, 7].

Cross-reactivity is less common to streptomycin, found to be 1–5% on patch testing [1]. Therefore, deoxystreptamine-sensitive individuals are less likely to develop allergic cross-reactions to streptomycin therapy.

#### Evaluation

Aminoglycoside hypersensitivity is relatively uncommon, and evaluation is often warranted only if there is an imminent need for treatment with this class of antibiotics, a clinical scenario most often encountered in patients with cystic fibrosis [25].

##### Immediate-Type Skin Testing

There are no validated skin tests for the diagnosis of immediate hypersensitivity to aminoglycosides. There are case reports of patients who experienced immediate clinical hypersensitivity reactions to gentamicin, tobramycin, neomycin, and streptomycin in which IgE-mediated allergy was confirmed by positive SPT [19–21, 23]. If SPTs are negative, intradermal tests (IDTs) can be performed with nonirritating concentrations. Nonirritating concentrations have been established for gentamicin and tobramycin and are shown in Table 1 [16, 26–28]. For SPTs and IDTs to streptomycin, cases have used 0.1–1 mg/mL initially, potentially increasing if negative to concentrations as high as 20 mg/mL; however, the irritant properties of higher concentrations have not been studied [28]. A cautious approach must be taken when evaluating anaphylactic reactions to streptomycin since systemic reactions have been observed after SPTs [1, 22]. Larger studies are needed to better determine the optimal concentration for skin testing of aminoglycosides and the negative and positive predictive value of these tests.

**Table 1:** Reported concentrations utilized for antibiotic skin testing. Concentrations have been established as nonirritating for gentamicin, tobramycin, and clindamycin

	**Full-strength concentrations commercially available in the USA**	**Nonirritating concentration**
Gentamicin [26–28]	40 mg/ml	4 mg/ml (1:10)
Tobramycin [16, 28]	40 mg/ml	4 mg/ml (1:10)
Clindamycin [27]	150 mg/ml	15 mg/ml (1:10)

##### Patch Testing

Patch testing involves application of non-irritating drug concentrations within a soluble vehicle such as petrolatum or water to the skin for 48 h to detect delayed hypersensitivity reactions. Patch testing can be useful in patients with contact dermatitis reactions. Neomycin is one of the most common contact allergens and is included in commercial patch test panels [28]. The concentration often used is 20% in petrolatum for neomycin, gentamicin, and tobramycin, and 1% in petrolatum for streptomycin [1]. In patients with suspected allergic contact dermatitis, patch test panels revealed neomycin as the offending agent in 2.5 to 3.6% of patients [1]. However, neomycin contact allergy has been reported with an even higher prevalence in some case series [29].

#### Management/Desensitization

Due to the infrequent nature of aminoglycoside hypersensitivity and availability of alternative agents in most cases, desensitization procedures are not generally performed. Case reports have demonstrated success when utilized for patients with cystic fibrosis with IgE-mediated hypersensitivity to tobramycin requiring IV or inhaled tobramycin for severe infections without suitable alternatives.

Protocols for IV tobramycin desensitization have been reported. Earl and Sullivan described tobramycin desensitization in a pediatric patient receiving treatment for a lung abscess who experienced generalized urticaria to both IV and inhaled tobramycin (Table 2) [16]. Eight patients within the Adult Cystic Fibrosis Unit at St. James’s Hospital in Leeds, UK, underwent a 7-step rapid IV tobramycin desensitization protocol with tenfold increases in concentration, each step over 20 min, until the therapeutic dose was achieved. This was successful in 7 patients, although the 8th patient felt unwell and developed fever during desensitization and was unable to complete the full desensitization protocol [30].

**Table 2:** Intravenous tobramycin desensitization protocol for immediate hypersensitivity [16]

Step	Dose (mg)	Cumulative dose (mg)
1	0.001	0.001
2	0.002	0.003
3	0.004	0.007
4	0.008	0.015
5	0.016	0.031
6	0.032	0.063
7	0.064	0.127
8	0.128	0.255
9	0.256	0.511
10	0.512	1.023
11	1	2.023
12	2	4.023
13	4	8.023
14	8	16.023
15	16	32.023
16	32	64.023
17	16	80.023

Each dose was in 20 mL of normal saline and infused over 20 min. Ten minutes after each infusion was completed, the subsequent dose was given, for a total of 8 h

An inhaled tobramycin desensitization protocol has also been performed successfully in a pediatric patient with cystic fibrosis pulmonary exacerbation [17] (Table 3).

**Table 3:** Inhaled tobramycin desensitization protocol for immediate hypersensitivity [17]

Step	Dose (mg)
1	0.3
2	0.6
3	0.9
4	1.2
5	1.5
6	3
7	6
8	12
9	24
10	48
11	96
12	150
13	200
14	250
15	300

Each dose was in 5 mL of normal saline and given via nebulizer every 2 h

## Clindamycin

### Overview

#### **Structure**:

**Fig. 2:**
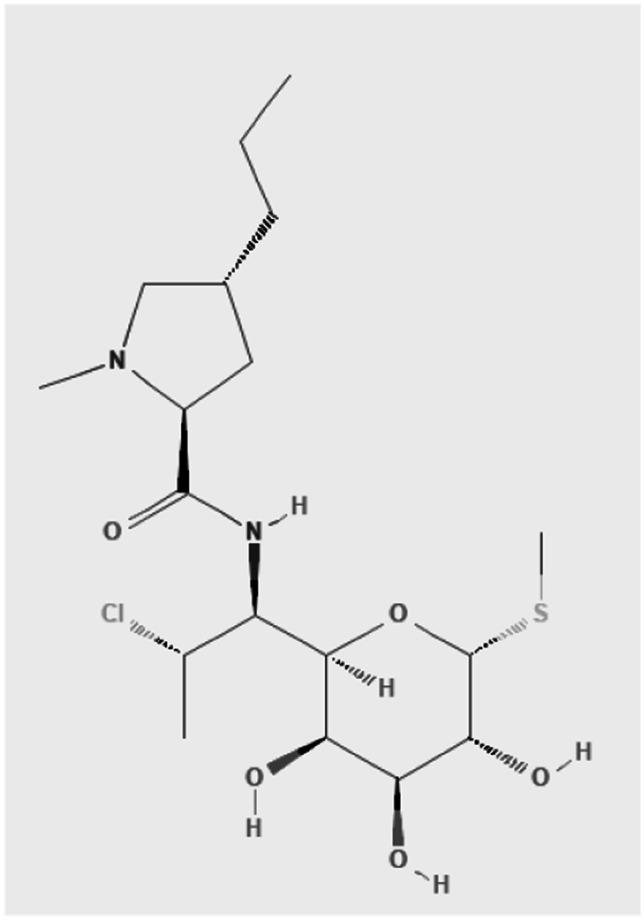
Chemical structure of clindamycin [31]

 Clindamycin is a derivative of lincomycin with a 7(S)-chloro-substitution of the 7(R)-hydroxyl group [31] (Fig. 2).

#### **Mechanism**:

 Clindamycin is bacteriostatic and inhibits bacterial protein synthesis by binding to the 50S ribosomal subunit [31].

#### **Indications**:

 Clindamycin is used in the treatment of respiratory, bone and soft tissue, neck, facial, abdominal, and pelvic infections caused by Gram-positive bacteria, including strains of MRSA, most anaerobic bacteria, and certain protozoa [1, 31].

#### **Known Pharmacologic Adverse Reactions**:

 The most common adverse effect associated with clindamycin is diarrhea. Clindamycin is frequently implicated in antibiotic-associated diarrhea due to Clostridium difficile colitis which can cause pseudomembranous colitis. Due to these increased risks, the use of clindamycin is often restricted to patients with severe infections or to patients with beta-lactam hypersensitivity. Some other adverse effects include metallic taste in the mouth, transient elevations in liver transaminases, granulocytopenia, and thrombocytopenia [1, 31].

### Hypersensitivity Reactions

#### Delayed Maculopapular Exanthem

The most common type of hypersensitivity reaction to clindamycin is a delayed maculopapular rash usually 7–10 days after initiation of the drug [1, 32]. Various case reports describe maculopapular skin eruptions with clindamycin [33–36]. Studies in the 1970s reported an incidence of rashes with clindamycin in approximately 10% of patients [37]. A more recent and much larger study of 3896 clindamycin administrations from a single US hospital reported a likely more realistic incidence of 0.47% with most of the rashes as delayed cutaneous reactions [38].

#### IgE-Mediated Reactions

Type I IgE-mediated hypersensitivity and anaphylactic reactions to clindamycin are rare with just a few cases described in the literature [39–41]. Bulloch et al. describe a patient with hypotension, confusion, dyspnea, and copious oral secretions 3 min after a dose of IV Clindamycin for a periodontal abscess [39]. Ebo et al. report a case of perioperative clindamycin anaphylaxis with localized hives along the infusion site, hypotension, hypoxia, and vomiting within 5 min after antibiotic administration [40]. Chiou et al. describe a case of clindamycin anaphylaxis during general anesthesia with sudden bronchospasm and hypotension which progressed to pulseless electrical activity necessitating cardiopulmonary resuscitation [41].

#### Other Hypersensitivity Reactions

Other immunologic drug reactions that have been reported include fixed drug eruptions [42]. Other rare hypersensitivity reactions to clindamycin include DRESS/DiHS [43–45], SDRIFE [46], AGEP [47–53], and acute febrile neutrophilic dermatosis or Sweet Syndrome [54–56].

DRESS is a rare delayed hypersensitivity reaction often occurring 2–8 weeks from drug exposure and characterized by skin eruption, fever, eosinophilia, lymphadenopathy, and internal organ (i.e., liver, kidney, lung) involvement [43–45]. DRESS is most commonly caused by aromatic anticonvulsants (i.e., phenobarbital, carbamazepine, phenytoin), lamotrigine, and sulfonamides, but a few case reports diagnosed DRESS in the setting of clindamycin [43–45].

AGEP is an uncommon cutaneous hypersensitivity reaction with diffuse, pinpoint, sterile, non-follicular pustules overlying erythematous skin with systemic manifestations including leukocytosis with neutrophilia, fever, transaminitis, and renal insufficiency [47]. AGEP is usually drug-induced and often related to antibiotics such as beta-lactams or macrolides. Historically, AGEP has rarely been associated with clindamycin. There have been increasing numbers of case reports of AGEP with clindamycin in the recent literature [47–53].

Acute febrile neutrophilic dermatosis or Sweet syndrome characterized by fever, leukocytosis with neutrophilia, and tender erythematous skin lesions with histologically dense dermal neutrophilic infiltration. This rare disorder is often idiopathic but can be associated with autoimmune and inflammatory disorders, malignancy-associated, or drug-induced. The most commonly associated drug is granulocyte-colony stimulating factor (GCSF) which stimulates the production of neutrophils, but case studies on a wide variety of drugs have been described including a few associated with clindamycin [54–56].

#### Cross Reactivity

There are no data on potential cross-reactivity of clindamycin with other antibiotics.

### Evaluation

#### Immediate-Type Skin Testing

Nonirritating concentrations have been established and are shown in Table 1 [26]. However, there are limited data regarding the diagnostic value of SPT and IDT in evaluating clindamycin hypersensitivity. Studies have shown that clindamycin skin testing is not useful in evaluating delayed maculopapular rashes, as would be expected with a non-IgE-mediated, T cell-mediated delayed hypersensitivity reaction [57, 58]. Among 13 patients with delayed reactions, none of the patients had a positive SPT although 3 of the patients had a positive oral challenge [58]. Among 14 patients with histories suggestive of immediate reactions, none of the patients had a positive SPT although 6 of the patients had a positive oral challenge with immediate reactions [58]. Two of the 31 patients had delayed reactions of erythema, edema, and pruritus that occurred after 72 from the clindamycin intradermal skin testing [58].

#### Patch Testing

Patch testing can be helpful for delayed cutaneous reactions that involve T cell-mediated hypersensitivity, particularly in patients with several potential culprit drugs. Patch testing with clindamycin, however, has yielded mixed results with sensitivity ranging between 15 and 30% [59, 60] but can be a non-invasive first step in evaluating possible delayed hypersensitivity reactions to clindamycin [59, 60]. Case studies have used clindamycin concentrations of 10 and 30% in petrolatum for patch testing [59, 60]. Given the high likelihood of false negative results on patch testing, careful monitoring is required when reintroducing the antibiotic since patch testing might not be enough to confirm absence of delayed reactions [1].

#### Lymphocyte Transformation Test

Cases of delayed allergic reaction to clindamycin have been confirmed with positive in-vitro lymphocyte transformation tests (LTTs) [43, 61]. LTT is sometimes used clinically in other countries but mostly used as a research tool in the USA.

### Management/Desensitization

Most cases of clindamycin delayed maculopapular exanthems do not require treatment and spontaneously resolve with cessation of the drug [1].

As immediate reactions to clindamycin are exceedingly rare, desensitization is rarely necessary. Desensitization could be utilized for patients who are determined to have immediate hypersensitivity to clindamycin either due to convincing history or positive testing. A rapid oral clindamycin desensitization has been successfully performed for a pediatric patient using a 9-step approach over 4.5 h until a cumulative dose of 300 mg of clindamycin was given [62] (Table 4).

**Table 4:** Oral clindamycin desensitization protocol for immediate hypersensitivity [62]

Step	Dose (mg)	Concentration (mg/mL)	Volume (mL)
1	0.005	0.02	0.25
2	0.05	0.02	2.5
3	0.5	0.2	2.5
4	5	2.0	2.5
5	10	20	0.5
6	20	20	1
7	40	20	2
8	80	20	4
9	150	Oral capsule	N/A

Intervals between doses were 30 min, for a total of 4.5 h

## Linezolid

### Overview

#### **Structure**:

**Fig. 3:**
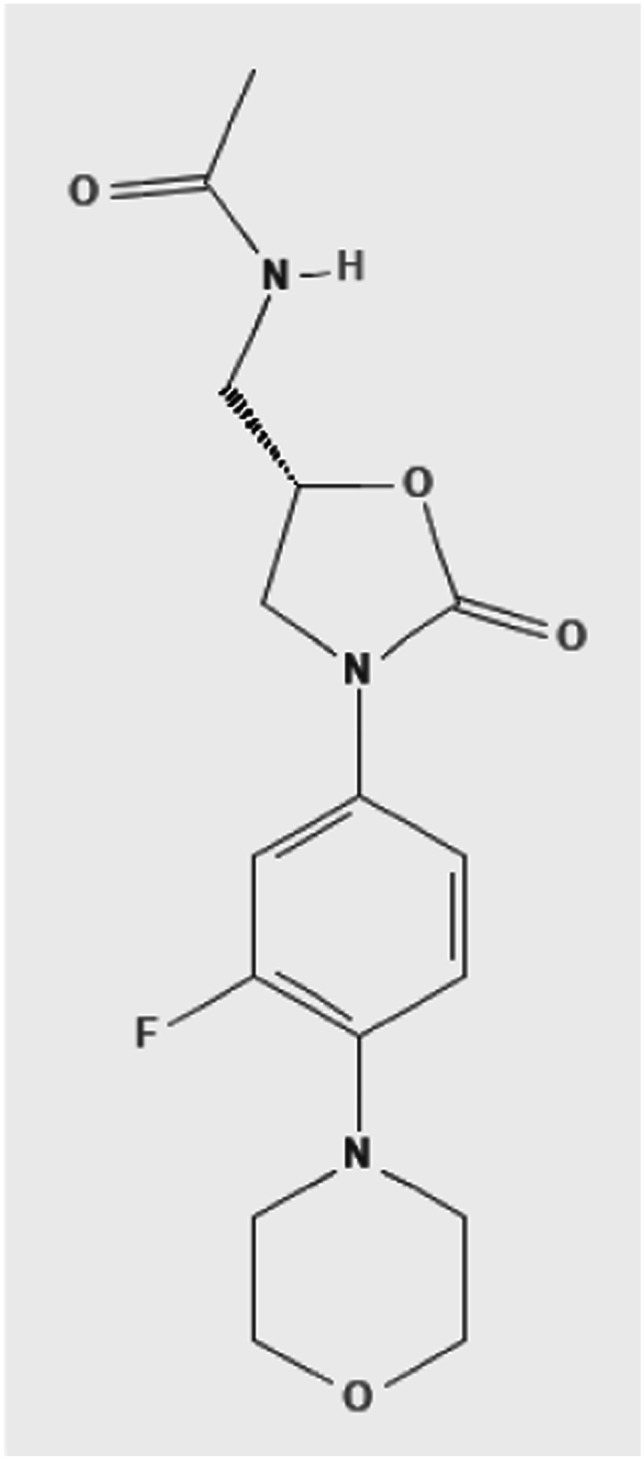
Chemical structure of linezolid [63]

 Linezolid is an oxazolidinone, a heterocyclic molecule with a nitrogen and oxygen in a 5-membered ring bridged with a carbonyl group [63] (Fig. 3).

#### **Mechanism**:

 Linezolid inhibits protein synthesis by binding to the 50S ribosomal subunit and preventing the formation of the 70S initiation complex. Linezolid is bactericidal against Streptococci and bacteriostatic against Staphylococci and Enterococci [63].

#### **Indications**:

 Linzolid is indicated for Gram-positive infections, especially clinically important for resistant microorganisms such as methicillin-resistant Staphylococcus aureus (MRSA) and vancomycin-resistant Enterococcus (VRE). Linezolid is used for bacterial pneumonia, bacteremia, bone/joint infections, and complicated/uncomplicated skin/soft tissue infections [63].

#### **Known Pharmacologic Adverse Reactions**:

 The most common adverse effects include diarrhea, headache, and nausea. Anemia, leukopenia, pancytopenia, and thrombocytopenia may occur in patients who are at risk for myelosuppression and who receive regimens > 2 weeks. Pseudomembranous colitis and neuropathy have also been reported. Serotonin syndrome can occur, and concurrent use is contraindicated with many anti-depressants [63].

### Hypersensitivity Reactions

Hypersensitivity reactions rarely occur with linezolid.

#### IgE-Mediated Reactions

Immediate hypersensitivity reactions to linezolid have been reported with symptoms including urticaria, skin flushing, and angioedema [64–66]. In one study, rash and pruritus were reported in 1.7% of the 828 patients who received linezolid as well as two cases of anaphylactoid-type reactions (one with bullous arm lesions and one with throat swelling) [64]. Bishop et al. reported that 1 of 44 patients experienced a severe skin rash from linezolid therapy that required drug discontinuation [65]. Yang and Xu report a case of a patient with angioedema and urticaria 12 h after initiation of linezolid for an Enterococcus faecium infection [66].

#### Other Hypersensitivity Reactions

There was a case of a patient with diffuse confluent non-blanching petechiae and purpura 9 days after starting linezolid with a punch biopsy showing a perivascular inflammatory infiltrate without noted changes of leukocytoclastic vasculitis [67]. There have also been case reports of linezolid-associated reactions such as interstitial nephritis and DRESS [68].

#### Cross Reactivity

There are no data on potential cross-reactivity of linezolid with other antibiotics.

### Evaluation

There are currently no standardized skin testing reagents or in vitro assays for assessment of linezolid-specific hypersensitivity. There are no reports of skin testing for evaluation of immediate reactions to linezolid.

### Management/Desensitization

There are a few cases of linezolid desensitization described in the literature. The protocol by Bagwell et al. is highlighted in Table 5 [69]. Guvenir et al. used a similar protocol as Bagwell et al. with the same 3 diluted solutions of linezolid but over a total of 13 steps (2 doses from the 0.02 mg/mL solution, 4 doses from the 0.2 mg/mL solution, and 7 doses from the 2 mg/mL) until the goal dose of 10 mg/kg was reached [70]. Cawley and Lipka treated a patient with multidrug-resistant Enterococcus faecium bacteremia by oral desensitization using an IV form of the drug. Due to lack of IV access and because of limited availability of the oral suspension from the manufacturer, a desensitization protocol was implemented in which the IV formulation of linezolid was given orally. The patient was successfully desensitized using an escalating, 14-dose procedure [71]. In all of these cases, the patients were able to successfully take linezolid without any reaction.

**Table 5:** Intravenous linezolid desensitization protocol for immediate hypersensitivity [69]

Step	Dose (mg)	Concentration (mg/mL)	Volume (mL)
1	0.03	0.02	1.5
2	0.07	0.02	3.5
3	0.15	0.2	0.75
4	0.3	0.2	1.5
5	0.6	0.2	3
6	1.2	0.2	6
7	2.3	2	1.2
8	4.7	2	2.3
9	9.4	2	4.7
10	18.75	2	9.4
11	37.5	2	18.8
12	75	2	37.5
13	150	2	75
14	300	2	150

Each dose was infused over 5 min except for the final 2 doses, which were infused over 15 min. Intervals between doses were 10 min

## Metronidazole

### Overview

#### **Structure**:

**Fig. 4:**
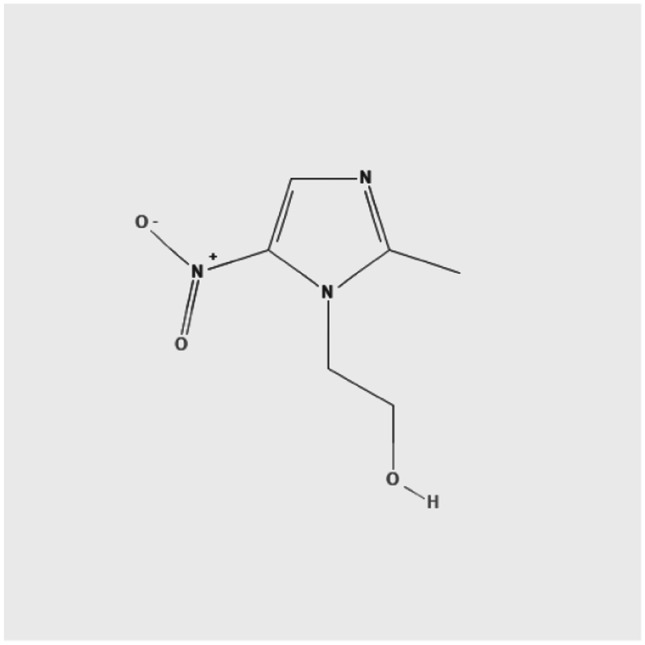
Chemical structure of metronidazole [72]

 Metronidazole is a nitroimidazole with structural similarities to tinidazole, clotrimazole, ketoconazole, miconazole, and albendazole [72] (Fig. 4).

#### **Mechanism**:

 Metronidazole is bactericidal. Metronidazole diffuses across cell membranes and is partially reduced by anaerobic bacteria and protozoa generating toxic free radicals and disrupting nucleic acid synthesis [72].

#### **Indications**:

 Metronidazole is one of the main drugs for the treatment of anaerobic infections and is also used for protozoal infections. It is used for gastrointestinal infections, Clostridium difficile, Helicobacter pylori, bacterial vaginosis, Trichomoniasis vaginalis, giardiasis, and amebiasis [72].

#### **Known Pharmacologic Adverse Reactions**:

 Some adverse effects of metronidazole include gastrointestinal symptoms, metallic taste, dark urine, dizziness, headaches, disulfiram-like reaction with alcohol, and hematological alterations [72].

### Hypersensitivity Reactions

Hypersensitivity reactions to metronidazole are rare with only a small number of case reports in the literature. However, a variety of different reaction types, both immediate and delayed, have been reported [8].

#### IgE-Mediated Reactions

Backus et al. report a patient experiencing severe itching and lip swelling during a second course of oral metronidazole for Trichomonas vaginalis infection [73]. Asensio et al. describe a patient with sneezing, rhinorrhea, perioral paresthesia, and upper airway angioedema followed by generalized pruritic erythematous lesions 30 min after spiramycin and metronidazole for gingivostomatitis [74]. The clinical history of positive skin prick test to metronidazole suggested anaphylaxis due to metronidazole [74].

#### Other Hypersensitivity Reactions

Other case reports include allergic contact dermatitis [75], fixed drug eruption [76–79], serum sickness-like reaction [80], SJS/TEN [81, 82], AGEP [83, 84], SDRIFE [85], and a possible case of DRESS given fever and rash but no lab studies were done to assess internal organ involvement [86].

Since metronidazole is available in various forms, sensitization may have occurred after topical application in some of the cases. For example, cases are described for patients using topical metronidazole gel for acne rosacea and experiencing allergic contact facial dermatitis [75].

#### Cross Reactivity

A potential for cross-reactivity exists between metronidazole and other imidazoles, such as tinidazole, clotrimazole, ketoconazole, miconazole, and albendazole [8, 87] given structural similarities [8]. For example, patch testing has demonstrated cross-reactivity between metronidazole and tinidazole [87].

### Evaluation

A small number of the case studies described positive skin testing to metronidazole correlating with patient history including positive SPT using 125 mg/mL [79] and patch testing varying from concentrations of 0.5 to 50% in petrolatum [75, 77, 79, 82, 84]. SPT was positive in one case of anaphylaxis [74] although other larger studies suggest that the sensitivity is low [79]. Given the low sensitivity of SPT, oral provocation is considered more useful for establishing the diagnosis in many cases of hypersensitivity reactions to metronidazole [79].

### Management/Desensitization

In patients at risk of hypersensitivity reactions to metronidazole without alternative treatment options, as may be the case for Trichomonas vaginalis, desensitization has been performed [8]. As there are no equally effective alternatives that have been systematically evaluated for the treatment of Trichomonas vaginalis, the CDC states that patients with an IgE-mediated reaction to a nitroimidazole may need to undergo metronidazole desensitization using published protocols [88]. Refer to Table 6 for the first published protocol by Kurohara et al. for oral metronidazole desensitization [89]. Table 7 shows a modified protocol for a more gradual dose escalation for cases with greater concern for severe systemic reactions [90]. Pearlman et al. devised an IV metronidazole desensitization protocol described in Table 8 [91]. In a case series of metronidazole desensitization among 15 patients, 8 use the oral protocol by Kurohara et al. and 7 use the IV protocol by Pearlman et al., and 1 patient in each group experienced a pruritic rash when reaching the goal dose. All 15 patients were cleared, and these studies help demonstrate efficacy of both oral and IV desensitization protocols for metronidazole.

**Table 6:** Oral metronidazole desensitization protocol for immediate hypersensitivity [89]

Step	Dose (mg)	Concentration (mg/mL)	Volume (mL)
1	0.0025	0.025	0.1
2	0.025	0.025	1
3	0.25	0.25	1
4	2.5	2.5	1
5	25	2.5	10
6	250	Oral tablet	N/A
7	500	Oral tablet	N/A
8	1000	Oral tablet	N/A

Time intervals not noted between doses

**Table 7:** Modified oral metronidazole desensitization protocol for immediate hypersensitivity [90]

Step	Dose (mg)	Concentration (mg/mL)	Volume (mL)
1	0.0025	0.025	0.1
2	0.025	0.025	1
3	0.25	0.25	1
4	2.5	2.5	1
5	5	2.5	2
6	10	2.5	4
7	25	2.5	10
8	50	25	2
9	100	25	4
10	250	Oral tablet	N/A
11	500	Oral tablet	N/A
12	1000	Oral tablet	N/A

Intervals between doses were 30 min

**Table 8:** Intravenous metronidazole desensitization protocol for immediate hypersensitivity [91]

Step	Dose (mg)	Concentration (mg/mL)	Volume (mL)
1	0.005	0.005	1
2	0.015	0.005	3
3	0.05	0.05	1
4	0.15	0.05	3
5	0.5	0.5	1
6	1.5	0.5	3
7	5	5	1
8	15	5	3
9	30	5	6
10	60	5	12
11	125	5	25
12	250	Oral tablet	N/A
13	500	Oral tablet	N/A
14	2000	Oral tablet	N/A

Intervals between intravenous doses were 15–20 min, and intervals between the final oral doses were 1 h

## Discussion

Aminoglycosides, clindamycin, linezolid, and metronidazole rarely cause drug hypersensitivity reactions, and there are only a few individual case reports of IgE-mediated systemic reactions to these antibiotics. Evaluation and management of possible hypersensitivity to these antibiotics is not recommended routinely but rather in situations when treatment with the drug is required and alternate agents cannot be substituted [26]. Refer to Table 9 for general considerations for antibiotic alternatives [92].

**Table 9:** Considerations for antibiotic alternatives [92]

Antibiotic	Possible alternatives
Aminoglycosides	• Pseudomonas aeruginosa: cefepime, ceftazidime, ciprofloxacin, piperacillin/tazobactam, meropenem/imipenem • Acinetobacter: TMP/SMX, ceftriaxone/cefotaxime (in addition to above) • Enterobacter, Citrobacter, or Serratia: cefuroxime (in addition to above) • Klebsiella: cefazolin/cephalexin (in addition to above) • Escherichia coli or Haemophilus influenzae: ampicillin, amoxicillin/clavulanate (in addition to above)
Clindamycin	• MRSA or CoNS: vancomycin, linezolid, daptomycin, ceftaroline • MSSA: cefazolin/cephalexin, oxacillin (in addition to above) • Streptococcus pneumoniae or pyogenes: penicillin, ampicillin, amoxicillin/clavulanate (in addition to above) • Anaerobic streptococci: penicillin, ampicillin, amoxicillin/clavulanate, cefazolin, cefoxitin, ceftriaxone/cefotaxime, meropenem/imipenem, piperacillin/tazobactam, metronidazole, vancomycin • Bacteroides fragilis: penicillin, ampicillin, amoxicillin/clavulanate, cefoxitin, meropenem/imipenem, piperacillin/tazobactam, metronidazole • Clostridia tetani or perfringens: penicillin, ampicillin, amoxicillin/clavulanate, cefoxitin, ceftriaxone/cefotaxime, meropenem/imipenem, piperacillin/tazobactam, metronidazole, vancomycin
Linezolid	• MRSA or CoNS: vancomycin, clindamycin, daptomycin, ceftaroline • MSSA: cefazolin/cephalexin, oxacillin (in addition to above) • Streptococcus pneumoniae or pyogenes: penicillin, ampicillin, amoxicillin/clavulanate (in addition to above) • Enterococcus faecalis or faecium: penicillin, ampicillin, amoxicillin/clavulanate, vancomycin, daptomycin
Metronidazole	• Clostridium difficile: vancomycin, meropenem/imipenem • Clostridia tetani or perfringens: penicillin, ampicillin, amoxicillin/clavulanate, cefoxitin, ceftriaxone/cefotaxime, meropenem/imipenem, piperacillin/tazobactam, clindamycin, vancomycin • Bacteroides fragilis: penicillin, ampicillin, amoxicillin/clavulanate, cefoxitin, meropenem/imipenem, piperacillin/tazobactam, clindamycin • Anaerobic streptococci: penicillin, ampicillin, amoxicillin/clavulanate, cefazolin, cefoxitin, ceftriaxone/cefotaxime, meropenem/imipenem, piperacillin/tazobactam, clindamycin, vancomycin

*MRSA* methicillin-resistant staphylococcus aureus, *CoNS* coagulase-negative staphylococcus aureus

General considerations for alternative antibiotics with similar coverage; however, alternatives largely depend on specific infection, susceptibilities, and possible toxicities. Infectious disease recommendations are useful for individual cases

### Immediate Reaction Evaluation/Management

There are no validated diagnostic tests for evaluation of IgE-mediated hypersensitivity to these antibiotics. Skin testing with nonirritating concentrations of the drug may provide useful information. However, the skin testing for the drugs discussed in this review are not standardized or validated and the positive and negative predictive values are largely unknown. There is also no validated serum IgE testing for these antibiotics. Additionally, tryptase levels in the acute phase of anaphylactic reactions have not been studied for these specific antibiotics. Oral challenge in a controlled and monitored environment may be useful for provoking and diagnosing hypersensitivity reactions to these antibiotics. However, it is contraindicated to re-administer drugs that caused SCARs such as SJS/TEN and DRESS/DiHS [26]. For patients with IgE-mediated hypersensitivity to these antibiotics for whom there are no alternative treatments, there are published desensitization protocols that can be utilized to promote a state of temporary tolerance.

### Delayed Reaction Evaluation/Management

Patch testing can be useful to evaluate delayed cutaneous reactions, and patch testing concentrations have been reported for aminoglycosides, clindamycin, and metronidazole. Patch testing is particularly relevant for topical aminoglycosides, especially neomycin, for which allergic contact dermatitis is the most common hypersensitivity reaction reported. Desensitization protocols, as with most other medications, have not been reported as a management strategy for delayed cutaneous reactions.

## Summary and Conclusions

In summary, aminoglycosides, clindamycin, linezolid, and metronidazole infrequently cause hypersensitivity reactions. Allergic contact dermatitis is the most frequent reaction associated with aminoglycosides, particularly topical neomycin for which patch testing can be useful and is validated. Clindamycin is most commonly associated with delayed maculopapular exanthems for which patch testing may be helpful but is not validated. IgE-mediated reactions and anaphylaxis to these antibiotics are uncommon with only a few reported cases and no validated immediate-type skin tests. There are rare case reports of DRESS/DiHS with aminoglycosides, clindamycin, linezolid, and metronidazole; AGEP with clindamycin and metronidazole; and acute febrile neutrophilic dermatosis with clindamycin. In situations when there are no suitable alternatives for an antibiotic that caused an immediate hypersensitivity reaction, desensitization protocols have been published for intravenous and inhaled tobramycin, oral clindamycin, intravenous linezolid, and oral and intravenous metronidazole that could be utilized to allow safe reintroduction of the antibiotic.

## References

[1] Sánchez-Borges M, Thong B, Blanca M (2013). Hypersensitivity reactions to non beta-lactam antimicrobial agents, a statement of the WAO special committee on drug allergy. World Allergy Organ J.

[2] Krause KM, Krause KM, Serio AW, Kane TR, Connolly LE (2016) Aminoglycosides: an overview.” Cold Spring Harb Perspect Med. 10.1101/cshperspect.a027029 10.1101/cshperspect.a027029 PMC4888811 27252397

[3] National Center for Biotechnology Information (2020) PubChem Compound Summary for CID 19649, Streptomycin. Retrieved from https://pubchem.ncbi.nlm.nih.gov/compound/Streptomycin

[4] National Center for Biotechnology Information (2020) PubChem Compound Summary for CID 3467, Gentamicin. Retrieved from https://pubchem.ncbi.nlm.nih.gov/compound/Gentamicin

[5] National Center for Biotechnology Information (2020) PubChem Compound Summary for CID 36294, Tobramycin. Retrieved from https://pubchem.ncbi.nlm.nih.gov/compound/Tobramycin

[6] National Center for Biotechnology Information (2020) PubChem Compound Summary for CID 8378, Neomycin. Retrieved from https://pubchem.ncbi.nlm.nih.gov/compound/Neomycin

[7] Childs-Kean LM, Shaeer KM, Varghese Gupta S, Cho JC (2019) Aminoglycoside allergic reactions. Pharmacy (Basel, Switzerland). 10.3390/pharmacy7030124 10.3390/pharmacy7030124 PMC6789510 31470509

[8] Chastain DB, Hutzley VB, Parekh J, Alegro JV (2019) Antimicrobial desensitization: a review of published protocols. Pharm: J Pharm Educ Practice. 10.3390/pharmacy7030112 10.3390/pharmacy7030112 PMC6789802 31405062

[9] De Pádua MCA, Uter W, Schnuch A (2007). Contact allergy to topical drugs: prevalence in a clinical setting and estimation of frequency at the population level. Pharmacoepidemiol Drug Saf.

[10] De Pádua MCA, Schnuch A, Lessmann H, Geier J, Pfahlberg A, Uter W (2005). Contact allergy to neomycin sulfate: results of a multifactorial analysis. Pharmacoepidemiol Drug Saf.

[11] Gilissen L, De Decker L, Hulshagen T, Goossens A (2019). Allergic contact dermatitis caused by topical ophthalmic medications: keep an eye on it. Contact Derm.

[12] González-Mendiola MR, Balda GA, Delgado CM, Montaño PP, De Olano GD, Sánchez-Cano M (2005). Contact allergy from tobramycin eyedrops. Allergy.

[13] Corazza M, Forconi R, Toni G, Scuderi V, Mantovani L, Borghi A (2019). Systemic allergic dermatitis due to gentamicin. Contact Derm.

[14] De Groot AC, Maibach HI (2010). Frequency of sensitization to common allergens: comparison between Europe and the USA. Contact Derm.

[15] Elliott JF, Abbas M, Hull P, De Gannes G, Toussi R, Milani A (2016). Decreasing rates of neomycin sensitization in western Canada. J Cutan Med Surg.

[16] Earl HS, Sullivan TJ (1987). Acute desensitization of a patient with cystic fibrosis allergic to both beta-lactam and aminoglycoside antibiotics. J Allergy Clin Immunol.

[17] Spigarelli MG, Hurwitz ME, Nasr SZ (2002). Hypersensitivity to inhaled TOBI® following reaction to gentamicin. Pediatr Pulmonol.

[18] Santos RP, Awa E, Anbar RD (2007). Inhaled tobramycin solution-associated recurrent eosinophilia and severe persistent bronchospasm in a patient with cystic fibrosis: a case report. BMC Pediatr. 10.1186/1471-2431-7-11 10.1186/1471-2431-7-11 PMC1820779 17331261

[19] Connolly M, McAdoo J, Bourke JF (2007). Gentamicin-induced anaphylaxis. Ir J Med Sci.

[20] Christiansen IS, Pedersen P, Krøigaard M, Mosbech H, Garvey LH (2016). Anaphylaxis to intravenous gentamicin with suspected sensitization through gentamicin-loaded bone cement. J Allergy Clin Immunol Pract.

[21] Henao CMG, Morales CIH, Villa RC, Henao AMC (2015). Gentamicin induced anaphylaxis, a case report. World Allergy Organ J.

[22] Jung D, Kim J, Choi NY (2014). Anaphylaxis associated with streptomycin skin testing. Ann Allergy Asthma Immunol.

[23] Añíbarro B, Seoane FJ (2009) Immediate allergic reaction due to neomycin. J Investig Allergol Clin Immunol. PMID: 19274933 19274933

[24] Bensaid B, Rozieres A, Nosbaum A, Nicolas J, Berard F (2012). Amikacin-induced drug reaction with eosinophilia and systemic symptoms syndrome: delayed skin test and ELISPOT assay results allow the identification of the culprit drug. J Allergy Clin Immunol.

[25] Parmar JS, Nasser S (2005). Antibiotic allergy in cystic fibrosis. Thorax.

[26] Solensky R, Khan DA (2010). Drug allergy: an updated practice parameter. Ann Allergy Asthma Immunol.

[27] Empedrad R, Darter AL, Earl HS, Gruchalla RS (2003). Nonirritating intradermal skin test concentrations for commonly prescribed antibiotics. J Allergy Clin Immunol.

[28] Solensky R (2020) Hypersensitivity reactions to macrolides, aminoglycosides, tetracyclines, clindamycin, and metronidazole. UpToDate. Accessed from https://www.uptodate.com/contents/hypersensitivity-reactions-to-macrolides-aminoglycosides-tetracyclines-clindamycin-and-metronidazole

[29] Yoo JY, Al Naami M, Markowitz O, Hadi SM (2010) Allergic contact dermatitis: patch testing results at Mount Sinai Medical Center. Skinmed. PMID: 21137633 21137633

[30] Whitaker P, Shaw N, Gooi J, Etherington C, Conway S, Peckham D (2011). Rapid desensitization for non-immediate reactions in patients with cystic fibrosis. J Cyst Fibros.

[31] National Center for Biotechnology Information (2020) PubChem Compound Summary for CID 446598, Clindamycin. Retrieved from https://pubchem.ncbi.nlm.nih.gov/compound/Clindamycin

[32] Rutkowski K, Wagner A, Mirakian R, Thomas I (2019). Immediate hypersensitivity to clindamycin: rare, but not impossible. J Allergy Clin Immunol Pract.

[33] Washington NR, Petersen K, Petersen M (2019). The clindamycin catastrophe: a case of antibiotic-induced skin eruption. Clin Pediatr.

[34] Monteagudo B, Cabanillas M, Iriarte P et al (2018) Clindamycin-induced maculopapular exanthema with preferential involvement of striae distensae: a koebner phenomenon? Acta Dermatovenerol Croat*.* PMID: 29782303 29782303

[35] Veraldi S, Guanziroli E, Ferrucci S, Nazzaro G (2019). Allergic contact dermatitis caused by clindamycin. Contact Derm.

[36] Romita P, Ettorre G, Corazza M, Borghi A, Foti C (2017). Allergic contact dermatitis caused by clindamycin mimicking ‘retinoid flare’. Contact Derm.

[37] Geddes AM, Bridgwater FAJ, Williams DN, Oon J, Grimshaw GJ (1970). Clinical and bacteriological studies with clindamycin. Br Med J.

[38] Mazur N, Greenberger PA, Regalado J (1999). Clindamycin hypersensitivity appears to be rare. Ann Allergy Asthma Immunol.

[39] Bulloch MN, Baccas JT, Arnold S (2016). Clindamycin-induced hypersensitivity reaction. Infection.

[40] Ebo DG, Mertens C, Braes M, Mennes I, Bridts CH, Sabato V (2019). Clindamycin anaphylaxis confirmed by in vivo and in vitro testing. J Allergy Clin Immunol Pract.

[41] Chiou C, Lin S, Lin S, Chang W, Chan K, Ting C (2006). Clindamycin-induced anaphylactic shock during general anesthesia. J Chin Med Assoc.

[42] Mahboob A, Haroon TS (1998). Drugs causing fixed eruptions: a study of 450 cases. Int J Dermatol.

[43] Nakamura Y, Watamatsu K, Muto M (2013). Drug-induced hypersensitivity syndrome induced by clindamycin. Acta Derm Venereol.

[44] Miller Quidley A, Bookstaver BP, Gainey AB, Gainey MD (2012). Fatal clindamycin-induced drug rash with eosinophilia and systemic symptoms (DRESS) syndrome. Pharmacotherapy.

[45] Tian D, Mohan RJ, Stallings G (2010). Drug rash with eosinophilia and systemic symptoms syndrome associated with clindamycin. Am J Med.

[46] Hernandez VC, Afonso MG, Viera AC, Martin-Fernandez L (2019). Symmetrical drug-related intertriginous and flexural exanthema due to clindamycin. BMJ Case Rep.

[47] Aiempanakit K, Apinantriyo B (2020). Clindamycin-induced acute generalized exanthematous pustulosis: a case report. Medicine.

[48] Croy C, Buehrle K, Szwak JA (2017). Clindamycin-associated acute generalized exanthematous pustulosis. J Clin Pharm Ther.

[49] De Cruz R, Ferguson J, Wee JS, Akhras V (2015). Acute localised exanthematous pustulosis (ALEP) induced by clindamycin in pregnancy. Australas J Dermatol.

[50] Kapoor R, Flynn C, Heald PW, Kapoor JR (2006). Acute generalized exanthematous pustulosis induced by clindamycin. Arch Dermatol.

[51] Smeets TJL, Jessurun N, Härmark L, Kardaun SH (2016) Clindamycin-induced acute generalised exanthematous pustulosis: five cases and a review of the literature. Neth J Med. PMID: 27966434 27966434

[52] Sulewski RJ, Blyumin M, Kerdel FA (2008) Acute generalized exanthematous pustulosis due to clindamycin. Dermatol Online J. PMID: 18718198 18718198

[53] Alniemi DT, Wetter DA, Bridges AG (2017). Acute generalized exanthematous pustulosis: clinical characteristics, etiologic associations, treatments, and outcomes in a series of 28 patients at Mayo Clinic, 1996–2013. Int J Dermatol.

[54] Kandula S, Burke WS, Goldfarb JN (2010). Clindamycin-induced sweet syndrome. J Am Acad Dermatol.

[55] Nitta K, Kano Y, Ushigome Y, Hayakawa J, Shiohara T (2019). Two cases of acute febrile neutrophilic dermatosis thought to be caused by topical clindamycin. Acta Derm Venereol.

[56] Clark BM, Homeyer DC, Glass KR, D’Avignon LC (2007). Clindamycin-induced sweet’s syndrome. Pharmacotherapy.

[57] Seitz CS, Bröcker E, Trautmann A (2009). Allergy diagnostic testing in clindamycin-induced skin reactions. Int Arch Allergy Immunol.

[58] Notman MJ, Phillips EJ, Knowles SR, Weber EA, Shear NH (2005). Clindamycin skin testing has limited diagnostic potential. Contact Derm.

[59] Pereira N, Canelas MM, Santiago F, Brites MM, Gonçalo M (2011). Value of patch tests in clindamycin-related drug eruptions. Contact Derm.

[60] Gilissen L, Huygens S, Goossens A, Breynaert C, Schrijvers R (2020). Utility of patch testing for the diagnosis of delayed-type drug hypersensitivity reactions to clindamycin. Contact Derm.

[61] Vílchez-Sánchez F, Domínguez-Ortega J, González Muñoz M et al (2020) Two case reports of delayed-allergic reactions to clindamycin confirmed with a positive lymphocyte transformation test. Eur Ann Allergy Clin Immunol. 10.23822/EurAnnACI.1764-1489.117. 10.23822/EurAnnACI.1764-1489.117 31668055

[62] Esty B, Minnicozzi S, Chu EC, Broyles AD, Yee CSK (2018). Successful rapid oral clindamycin desensitization in a pediatric patient. J Allergy Clin Immunol.

[63] National Center for Biotechnology Information (2020) PubChem Compound Summary for CID 441401, Linezolid. Retrieved from https://pubchem.ncbi.nlm.nih.gov/compound/Linezolid

[64] Birmingham MC, Rayner CR, Meagher AK, Flavin SM, Batts DH, Schentage JJ (2003). Linezolid for the treatment of multidrug-resistant, gram-positive infections: experience from a compassionate-use program. Clin Infect Dis.

[65] Bishop E, Melvani S, Howden BP, Charles PGP, Grayson LM (2006). Good clinical outcomes but high rates of adverse reactions during linezolid therapy for serious infections: a proposed protocol for monitoring therapy in complex patients. Antimicrob Agents Chemother.

[66] Yang M, Xu M (2012). Linezolid-induced angioedema and urticaria in a patient with renal failure. Braz J Infect Dis.

[67] Kim FS, Kelley W, Resh B, Goldenberg G (2009). Linezolid-induced purpuric medication reaction. J Cutan Pathol.

[68] Savard S, Desmeules S, Riopel J, Agharazii M (2009). Linezolid-associated acute interstitial nephritis and drug rash with eosinophilia and systemic symptoms (DRESS) syndrome. Am J Kidney Dis.

[69] Bagwell AD, Stollings JL, White KD (2013). Linezolid desensitization for a patient with multiple medication hypersensitivity reactions. Ann Pharmacother.

[70] Guvenir H, Dibek Misirlioglu E, Toyran M, Kocabas CN (2016). Linezolid desensitization in a pediatric patient. Ann Allergy Asthma Immunol.

[71] Cawley MJ, Lipka O (2006). Intravenous linezolid administered orally: a novel desensitization strategy. Pharmacotherapy.

[72] National Center for Biotechnology Information (2020) PubChem Compound Summary for CID 4173, Metronidazole. Retrieved from https://pubchem.ncbi.nlm.nih.gov/compound/Metronidazole

[73] Backus KV, Muzny CA, Beauchamps LS (2017). Trichomonas vaginalis treated with boric acid in a metronidazole allergic female. Sex Transm Dis.

[74] Asensio Sánchez T, Dávila I, Moreno E et al (2008) Anaphylaxis due to metronidazole with positive skin prick test. J Investig Allergol Clin Immunol. PMID: 18447147 18447147

[75] Madsen JT, Thormann J, Kerre S, Andersen KE, Goossens A (2007). Allergic contact dermatitis to topical metronidazole - 3 cases. Contact Derm.

[76] Kumar N, Sundriyal D, Walia M, Trisal D (2013). Metronidazole-induced fixed drug eruption. BMJ Case Rep.

[77] Prieto A, De Barrio M, Infante S, Torres A, Rubio M, Olalde S (2005). Recurrent fixed drug eruption due to metronidazole elicited by patch test with tinidazole. Contact Derm.

[78] Hermida MD, Consalvo L, Lapadula MM, Giovanna PD, Cabrera HN (2011). Bullous fixed drug eruption induced by intravaginal metronidazole ovules, with positive topical provocation test findings. Arch Dermatol.

[79] García-Rubio I, Martínez-Cócera C, Santos Magadán S, Rodríguez-Jiménez B, Vázquez-Cortés S (2006). Hypersensitivity reactions to metronidazole. Allergol Immunopathol.

[80] VanCleave HZ, Sanchez AC, Lieberman JA, Ellenburg JT, Mabry WQ (2016). Probable metronidazole induced serum sickness-like reaction in a paediatric patient. J Clin Pharm Ther.

[81] Mazumdar G, Shome K (2014). Stevens-Johnson Syndrome following use of metronidazole in a dental patient. Indian J Pharmacol.

[82] Piskin G, Mekkes JR (2006). Stevens-Johnson Syndrome from metronidazole. Contact Derm.

[83] Watsky KL (1999) Acute generalized exanthematous pustulosis induced by metronidazole: the role of patch testing. Archi Dermatol. 10.1001/archderm.135.1.93. Available on PubMed at: https://pubmed.ncbi.nlm.nih.gov/9923793/10.1001/archderm.135.1.93 9923793

[84] Kostaki M, Polydorou D, Adamou E, Chasapi V, Antoniou C, Stratigos A (2019). Acute localized exanthematous pustulosis due to metronidazole. J Eur Acad Dermatol Venereol.

[85] Sikar Aktürk A, Bayramgürler D, Salman S, Yıldız DK, Demirsoy EO (2014). Symmetrical drug-related intertriginous and flexural exanthema (SDRIFE) induced by oral metronidazole. Cutan Ocul Toxicol.

[86] Seto K, Knowles SR, Weber EA (2012). Immediate hypersensitivity reaction induced by metronidazole. Ann Pharmacother.

[87] Mishra D, Mobashir M, Zaheer SM (1990). Fixed drug eruption and cross-reactivity between tinidazole and metronidazole. Int J Dermatol.

[88] Helms DJ, Mosure DJ, Secor EW, Workowski KA (2008). Management of trichomonas vaginalis in women with suspected metronidazole hypersensitivity. Am J Obstet Gynecol.

[89] Kurohara ML, Kwong FK, Lebherz TB, Klaustermeyer WB (1991). Metronidazole hypersensitivity and oral desensitization. J Allergy Clin Immunol.

[90] Gendelman SR, Pien LC, Gutta RC, Abouhassan SR (2014). Modified oral metronidazole desensitization protocol. Allergy Rhinol.

[91] Pearlman MD, Yashar C, Ernst S, Solomon W (1996). An incremental dosing protocol for women with severe vaginal trichomoniasis and adverse reactions to metronidazole. Am J Obstet Gynecol.

[92] Bradley JS, Barnett ED, Cantey JB et al (2018) Preferred therapy for specific bacterial and mycobacterial pathogens. Nelson’s Pediatric Antimicrobial Therapy. Am Acad Pediatr 119–142

